# The effects of low doses of proton, iron or silicon radiation on spatial learning in a mouse model of Alzheimer's disease

**DOI:** 10.1093/jrr/rrt154

**Published:** 2014-03

**Authors:** John A. Bellone, Richard E. Hartman, Roman Vlkolinský

**Affiliations:** 1 Department of Psychology, Loma Linda University, 11130 Anderson Street, Suite 106, Loma Linda, CA 92350, USA; 2 Division of Radiation Research, Department of Basic Sciences, Loma Linda University, Loma Linda, CA, USA

**Keywords:** proton, iron, silicon, particle radiation, Alzheimer's disease, APP/PSEN1

## Abstract

Astronauts venturing outside low-Earth orbit risk exposure to charged particle radiation that can cause neurological deficits via oxidative stress, synaptic changes, altered neurogenesis and neuroinflammation. Because these responses are similar to those observed in age-related neurodegenerative diseases, we hypothesized that individuals with a propensity toward developing Alzheimer's disease (AD) would be more adversely affected by such exposure. To test this hypothesis, we exposed young double transgenic (tg) APP/PSEN1 mice (a commercially available strain engineered to develop AD-like neuropathology) and their wild-type (wt) counterparts to low doses of proton radiation (150 MeV/n, whole body). Spatial learning ability, a sensitive behavioral marker of hippocampal damage, was assessed using the water maze (WM) prior to exposure, then 3 and 6 months post-irradiation. Results showed that wt mice outperformed tg mice at each time-point, and irradiation of tg mice at doses 0.1, 0.5 and 1 Gy did not induce spatial learning deficits. However, deficits were observed in wt mice exposed to 0.5 Gy compared with 0 Gy wt controls at 6 months post-irradiation. Specifically, the 0.5 Gy group performed worse than the control group during the reversal learning phase of the WM, when the platform was switched to a new location (*P* < 0.02). The irradiated mice were unable to alter their search strategy and often perseverated on the previous platform location. Reduced reversal learning demonstrates cognitive inflexibility in novel situations and may be indicative of hippocampal memory system dysfunction.

In a follow-up study to assess the effects of high-LET particles on AD-like pathology, we exposed tg mice to low doses (0.1, 0.5 or 1 Gy) of either iron (600 MeV/n) or silicon (250 MeV/n) radiation and assessed the effects on spatial learning ability in the WM at 3 and 6 months post-irradiation. Mice exposed to 1 Gy of iron radiation performed better than 0 Gy tg controls in the WM at the 6 month time-point (*P* < 0.03). Conversely, mice exposed to 0.1 Gy of silicon radiation performed significantly worse than controls in the WM at the 3 month time-point (*P* < 0.02), but these deficits had ameliorated by 6 months. Mice exposed to 0.5 and 1 Gy of silicon radiation exhibited changes similar to those observed in the 0.1 Gy group, but the decrements did not reach statistical significance. These data suggest that exposure to low doses of iron radiation decreases the behavioral effects of AD-like neuropathology in the long-term, while silicon radiation exacerbates short-term cognitive impairment in individuals with a propensity for such pathology. Electrophysiological and immunohistochemical data are forthcoming, and will aid in elucidating potential mechanisms responsible for the observed effects.

Altogether, these results suggest that various types of particles induce different behavioral effects at low doses in APP/PSEN1 tg mice. Namely, proton radiation was found to have no effect on performance, iron radiation improved performance in the long-term and silicon radiation temporarily impaired performance. The finding that low doses of iron and silicon radiation alter performance on a hippocampus-dependent task could have implications for astronauts with a predisposition to developing AD-like neurodegenerative changes who travel on extended missions outside Earth's magnetosphere.

